# AI integration and workforce development: Exploring job autonomy and creative self-efficacy in a global context

**DOI:** 10.1371/journal.pone.0319556

**Published:** 2025-06-04

**Authors:** Deeviya Francis Xavier, Christian Korunka, Roni Reiter-Palmon

**Affiliations:** 1 Department of Occupational, Economic and Social Psychology, University of Vienna, Vienna, Austria,; 2 Department of Psychology, University of Nebraska Omaha, Omaha, United States of America; Instituto Tecnologico Autonomo de Mexico, MEXICO

## Abstract

This paper explores the relationship between Artificial Intelligence (AI) integration in the workplace, cultural orientation, and its impact on job autonomy and creative self-efficacy. Our study employs a mixed-method experimental design across 480 individuals from different cultural backgrounds, specifically individualistic (United Kingdom) and collectivistic (Mexico) cultures. We evaluate how they perceive AI’s role in their professional lives. We focus on two key aspects: job autonomy, the level of control and discretion employees have over their tasks, and creative self-efficacy, the confidence in one’s ability to generate innovative ideas. Our findings revealed a significant increase in job autonomy following AI integration across all participants. Interestingly, this increase was more pronounced in the individualistic participants. Regarding creative self-efficacy, we found gender-specific impacts, with male participants experiencing a decrease, contrary to our expectations. Finally, our results supported the hypothesis that cultural orientation influences perceptions of AI, with collectivistic participants being more receptive to AI integration. These findings have significant implications for organizations integrating AI in multicultural environments. They highlight the importance of considering cultural differences in AI deployment strategies and suggest a need for culturally sensitive AI systems. The study also opens avenues for future research, particularly in exploring the role of other cultural dimensions, conducting longitudinal studies, and investigating ethical and bias-related aspects of AI in the workplace.

## Introduction

Artificial Intelligence (hereafter, AI) has undergone a profound transformation across various industries, marking an era of advanced technological integration and unparalleled operational efficiency. The integration of AI into workplaces to automate mundane tasks has not only heightened efficiency but also redirected human workers towards intricate and creative endeavors [1,2,3]. For example, in customer service, AI chatbots adeptly manage routine inquiries, enabling human agents to address more complex issues [4]. In supply chain management, AI has transcended basic inventory tracking to predict disruptions and optimize logistics [5]. In the healthcare sector, AI algorithms have assumed a pivotal role in diagnostics and personalized treatment planning [6]. Meanwhile, the manufacturing sector relies on AI for predictive maintenance, reducing downtime and costs, and the finance industry utilizes AI for fraud detection and personalized banking services [7]. In terms of decision-making, AI provides unparalleled data-driven insights, particularly in marketing, where it is employed to analyze consumer data, tailor marketing strategies, and forecast trends [8].

In this study, we address the core research question: How does AI integration influence employees’ perceptions of job autonomy and creative self-efficacy, and do these effects differ by cultural orientation (individualistic versus collectivistic)? By focusing on Mexico and the United Kingdom, we capture meaningful contrasts in cultural orientations that allow us to examine how AI deployment might vary across these national contexts.

Initially, AI was confined to automating repetitive tasks. However, advancements in machine learning and deep learning have allowed AI systems to learn from data, refine algorithms, and make increasingly complex decisions [9, 10]. This evolution—from basic automation to advanced support systems—stands as a testament to AI integration’s growing significance across diverse sectors. Studies have shown that while AI could automate certain tasks, it also creates new tasks and roles, leading to a transformation of jobs rather than their elimination [11,12]. Bessen’s historical analysis of technological change in the workplace revealed that new technologies often require new skill sets, thus changing the nature of jobs rather than eradicating it [13]. Similarly, a study by Davenport and Ronanki focused on the concept of “augmented intelligence,” where they emphasized the potential for AI to improve employee performance, particularly in areas requiring data analysis and interpretation [14].

In exploring the intersection of technology and cultural differences, Ji and colleagues delved into how employees from diverse cultural backgrounds perceive technology’s role in the workplace [15]. Their research revealed that individuals from collectivistic cultures tend to view technology advancements more favorably when it is seen as enhancing group collaboration. In contrast, those from individualistic cultures showed a preference for technology applications that bolster personal efficiency and autonomy.

The theories of individualism and collectivism, central to Hofstede’s cultural dimensions, offer a comparative lens to understand these variances in values, beliefs, and behaviors [16]. Individualism, with its emphasis on personal achievements, autonomy, and self-reliance, is predominant in societies like the United States and Western Europe, where personal goals and independence are highly valued. Conversely, collectivism emphasizes group goals, community ties, and collective well-being, a characteristic of many Asian and Latin American cultures, where the group’s needs and goals often supersede individual desires [17]. This dichotomy significantly impacts technology adoption and workplace behavior. In individualistic cultures, the drive for technology adoption is often linked to enhancing personal efficiency, innovation, and gaining a competitive edge [18]. Employees in these cultures may favor technologies that improve individual performance and offer avenues for personal and career development [19]. AI is often perceived as a tool to boost personal productivity and decision-making autonomy, with a keen interest in how it can facilitate independent work and skill development [4]. On the other hand, collectivistic cultures may approach technology adoption with a group-centric focus, valuing technologies that promote communication, collaboration, and group cohesion [20].

While existing research offers insights into the broader impacts of AI on technology adoption, there’s a lack of depth in exploring how these variables intersect and influence key dimensions of cultural orientations, employee well-being and productivity, namely job autonomy and creative self-efficacy [21]. Job autonomy was chosen as a central variable to reflect perceptions of AI integration because it is a well-established measure of employees’ sense of control and decision-making power in their roles, particularly within individualistic cultures where personal autonomy is highly valued [12,4,21,22,23]. Theoretical foundations for job autonomy, rooted in self-determination theory, suggest that autonomy is a critical factor for job satisfaction, motivation, and performance [21]. Autonomy in the workplace refers to the degree of control and discretion employees have over their tasks and the way they perform them [12]. In the context of AI integration, there is a concern that increased automation and decision-making by AI systems could undermine this sense of autonomy, particularly in individualistic cultures where personal control and independence are highly valued [4].

Creative self-efficacy, deeply rooted in Bandura’s social cognitive theory, is an essential variable for understanding the true effects of AI integration on employee behaviour, particularly in decision-making abilities [24]. According to Bandura, this concept relates to an individual’s belief in their capacity to produce creative outcomes [25]. In the workplace, this translates into the confidence employees have in their ability to generate innovative and effective solutions, a critical aspect of modern professional environments where adaptability and innovation are highly valued [19]. Individuals with high creative self-efficacy are more likely to take risks, explore unconventional approaches and persist against challenges, enhancing their performance in problem-solving and decision-making tasks [26,27]. The integration of AI in the workplace has a significant potential to enhance creative self-efficacy through AI’s ability to identify patterns and insights that might not be immediately apparent to human analysis, leading to more informed and innovative decisions [19].

However, the intersection of AI, job autonomy, and creative self-efficacy becomes even more complex and relevant when considered within different cultural contexts. Cultural factors play a crucial role in shaping these variables [17,28]. For instance, in collectivistic cultures, AI might be seen as a tool that enhances group collaboration and collective creative efforts, positively influencing self-efficacy in creative tasks. Conversely, in individualistic cultures, AI might be viewed as a means to bolster individual performance and decision-making capabilities [29, 18]. Therefore, assessing changes in creative self-efficacy in response to AI integration can provide valuable insights into how AI tools are influencing employee decision-making abilities. If AI integration correlates with an increase in creative self-efficacy, it suggests that AI tools are being effectively utilized to enhance these abilities. In contrast, a decrease in creative self-efficacy post-AI integration could indicate an over-reliance on AI, possibly undermining individuals’ confidence in their own decision-making skills.

This manuscript aims to investigate the complex interplay between AI integration and its impact on job autonomy and creative self-efficacy across diverse cultural contexts. Such an inquiry is essential, as it delves into the significant, though insufficiently explored, effects of AI on workplace dynamics across different cultural landscapes. Specifically, job autonomy and creative self-efficacy are chosen as key variables because they are deeply influenced by cultural contexts, making them ideal for examining the intersection of AI integration and cultural orientations [21,23]. Therefore, we propose the following hypotheses:

1. **AI Integration and Job Autonomy**: AI integration negatively influence perceived job autonomy.
2. **AI Integration and Creative Self-Efficacy**: AI integration positively influences creative self-efficacy.
3. **Cultural Orientation and Perception of AI Integration**: Individuals from collectivistic cultures are more likely to be receptive to AI integration compared to those from individualistic cultures.

By exploring these hypotheses, this study aims to deepen the understanding of how AI integration intersects with cultural orientation to impact job autonomy and creative self-efficacy.

## Materials and methods

### Study design

For the study design, we employed a methodological framework that integrates both between-subjects and within-subjects components. The between-subjects component of our study entailed the comparison of participants hailing from two culturally distinct backgrounds: the United Kingdom (hereafter, UK) and Mexico. The selection of Mexico and the UK for this study was strategically based on their respective scores on Hofstede’s Individualism index, which assesses individualism across 102 countries [30,17]. Mexico, with a score of 34, ranks among the lowest for individualism, aligning it closely with collectivist cultures. In contrast, the UK, scoring 76, ranks among the highest, positioning it firmly within individualistic cultures. It is noteworthy that both countries have recognized the importance of AI at the national policy level. The United Kingdom, for example, introduced its National AI Strategy – AI Action Plan, released in July 2022, which emphasizes responsible AI development and outlines initiatives for accelerating AI adoption [31]. Meanwhile, Mexico’s National AI Agenda, released in May 2024, details strategies to identify AI opportunities and address societal needs, indicating a growing commitment to incorporating AI into its economic landscape [32]. These differing national priorities may further shape employees’ attitudes toward AI integration in the workplace. By conducting separate analyses for each cultural group, we aimed to unveil overarching cultural differences in how AI integration is perceived and how it impacts job autonomy and creative self-efficacy. This approach allowed us to explore the macro-level variations that cultural orientation may introduce into these domains. Concurrently, the within-subjects component of our design delved into the effect of AI integration at the micro-level, focusing on job autonomy and creative self-efficacy within each cultural group. By employing a within-subjects design, we were able to assess how individual participants’ perceptions and attitudes were influenced when exposed to AI-integrated scenarios compared to non-AI-integrated ones. To ensure the integrity of our data, randomization was implemented to assign task scenarios to participants. This strategy minimized order effects and sequence biases, allowing each participant to encounter both scenarios—AI-integrated and non-AI-integrated. This approach prevents any potential bias that could arise from the order in which tasks are presented, thereby ensuring the reliability of our findings [33].

### Demographics of participants

**Fig 1:**
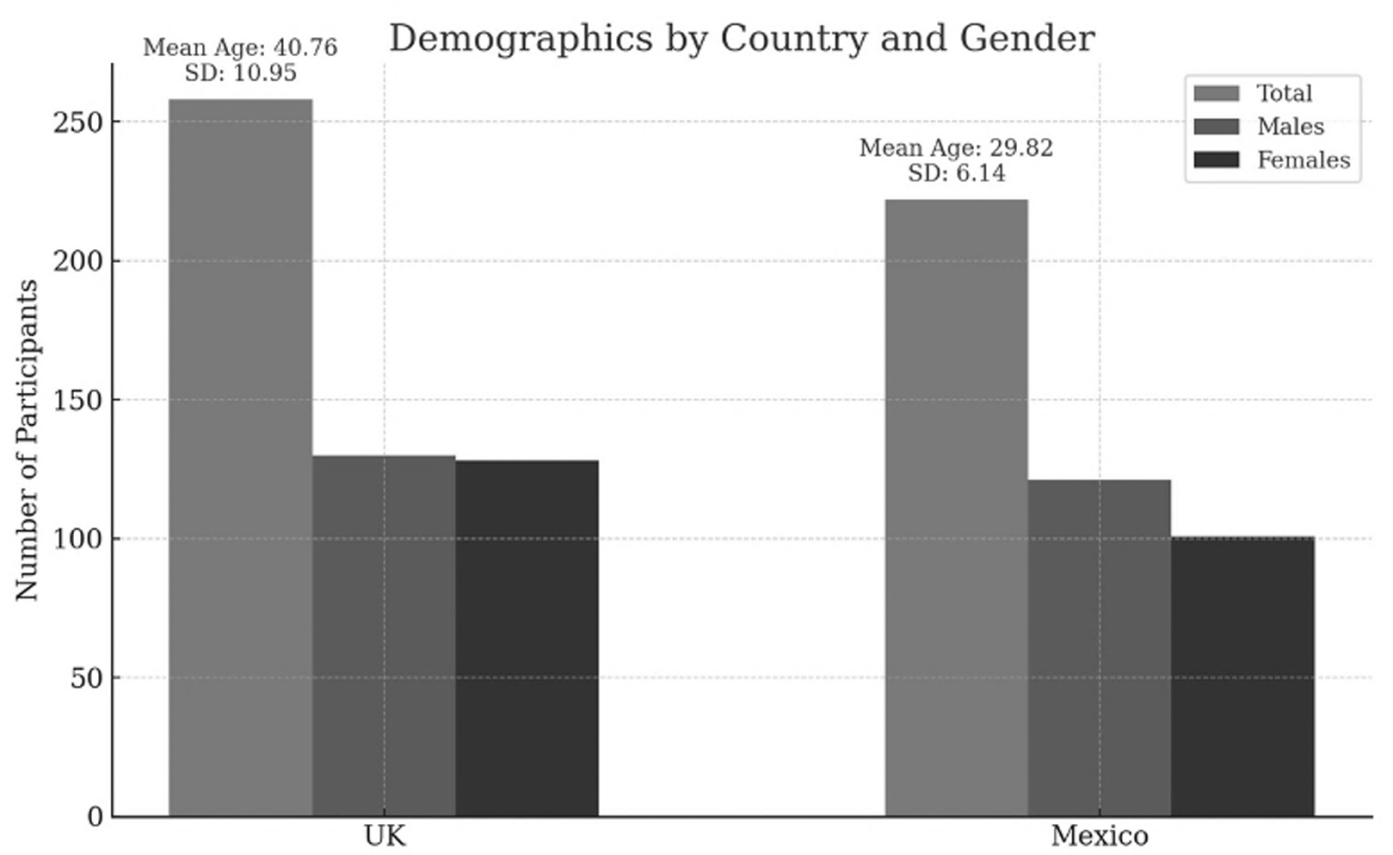
Demographics by Country and Gender.

In this section, we provide a more detailed description of the participants, highlighting the demographic characteristics that shed light on the diversity of our sample. The study engaged a total of 480 participants were evenly distributed between two culturally distinct groups: UK and Mexico, across the following sectors: Accounts and Finance, Business Strategy, Customer and Client Handling, Hiring, Marketing and Advertising, Operations and Production, People Management, Research and Development, and Supply Chain and Logistics. This deliberate focus on business roles underscores the relevance of the sample to our research objectives, which aim to explore AI’s implications in environments where its application is both critical and transformative. In the UK cohort, comprising 258 individuals, there was a balanced representation of genders, with 130 males and 128 females. The age range of participants in the UK spanned from 18 to 65 years, with a mean age (M) of 40.76 and a standard deviation (SD) of 10.95. Meanwhile the Mexican group, consisting of 222 participants, featured 121 males and 101 females. The age range of participants from Mexico was between 18 and 54 years, with a mean age (M) of 29.82 and a standard deviation (SD) of 6.14. (Fig 1) shows a visual representation of the demographics of the participants by country and gender.

### Data collection

Data collection was planned to align with the highest scientific standards and ethical considerations. The Institutional Review Board (IRB) of the Department of Applied Psychology: Work, Education, Economy at the University of Vienna granted approval (Approval Number: 2019/A/002), ensuring all procedures were ethically sound. Participant recruitment was conducted via Prolific. Upon confirming eligibility, participants were provided with detailed information about the study, titled “Decision-making Training,” which was deliberately named to obscure the true objective of examining how cultural orientations influence AI-integrated scenarios. This precaution was taken to avoid introducing bias into the participants’ responses. They were informed that the activity would involve participating in decision-making exercises and completing brief surveys before and after these exercises, with a reassurance that no sensitive personal information would be collected. Participants were also asked if they agreed with the conditions, and if they clicked ‘Disagree,’ the survey was set to end automatically. The total expected time commitment was outlined as approximately 20 minutes. Data collection commenced and concluded on November 13, 2023.

Participants were required to meet specific criteria: be 18 years or older, have a reliable internet connection, use a computer desktop, and reside in either the UK or Mexico. Consent was secured through Qualtrics, which was configured to terminate the survey for those who did not consent, thus upholding the principle of voluntary participation. Subsequent to obtaining consent, demographic questionnaires were administered to collect data on age, gender and professional background. This facilitated a thorough characterization of the sample’s diversity. Participants were then assessed using a cultural orientation scale to ensure accurate classification into cultural groups based on their individual scores, rather than mere geographical origin [28]. This ensured a precise alignment of participants with either collectivist or individualist orientations. Following this classification, participants were then randomly assigned to various decision-making scenarios, both with and without AI integration. Their decision outcomes and reflections on autonomy and creative self-efficacy were recorded. Upon survey completion, participants were debriefed and thanked for their contributions.

### AI integration exercise

The study included a two-part exercise to evaluate decision-making processes involving AI integration:

1. **Without AI Integration**: Participants analysed sales data and bar charts for four products, deciding on the allocation of an increased marketing budget to one product without the aid of AI. Their decisions were recorded for later analysis.
2. **With AI Integration**: Participants engaged in a similar task, but this time with AI-generated insights informing their decisions. These insights were provided by a text-based AI simulation using OpenAI’s ChatGPT, embedded within the Qualtrics survey platform. The AI, functioning as a language model, was programmed to analyse and interpret sales data presented in chart form and generate strategic recommendations. This setup allowed participants to directly compare their decision-making processes with and without AI assistance.

### Survey instruments

In addition to the experimental manipulation of AI integration, we employed the following survey scales to measure our key constructs. Participants completed these scales both before and after the decision-making exercises, enabling us to assess changes in job autonomy and creative self-efficacy.

- **AI Integration** was manipulated experimentally through two separate tasks: one without AI support and one with AI support. This design allowed us to assess changes in job autonomy and creative self-efficacy between non-AI and AI-assisted tasks, rather than relying on a self-report scale to gauge AI perceptions.
- **Cultural orientation** was assessed once at the beginning of the survey using a reduced version of the horizontal and vertical individualism and collectivism scale originally adapted from Hofstede’s model and further developed by Sivadas and colleagues. (Sample item for individualism: ‘I enjoy being unique and different from others in many ways.’. Sample item for collectivism: ‘I usually sacrifice my self-interest for the benefit of my group.’) [16,28].
- **Job Autonomy** was assessed using a modified version of the Job Autonomy Scale, originally proposed by Breaugh and Becker [12]. This scale has been extensively validated and employed in various organizational research studies. (Sample item: ‘I have considerable opportunity for independence and freedom in how I do my job’). Cronbach’s α = .89.
- **Creative Self-Efficacy** was measured using the Creative Self-Efficacy Scale, as developed by Tierney and colleagues [24]. This scale was chosen for its psychometric properties and its ability to discern the self-perceived creative capabilities of individuals. (Sample item: ‘I feel confident in my ability to propose novel ideas.’). Cronbach’s α = .91.

## Results

In this section, we present the findings of our study aimed at exploring the multifaceted impact of AI integration in the workplace.

### Hypothesis 1: AI integration and job autonomy

- **Original Hypothesis**: AI integration may negatively influence perceived job autonomy.
- **Overall Results**: Contrary to the original hypothesis, there was a **significant increase** in job autonomy after AI integration, with a t-statistic of **2.91**, p-value of **0.004**, and an **effect size** (Cohen’s d =  **0.12**). The 95% confidence interval (CI) for the effect size ranged from **0.04 to 0.20**.
- **Exploratory Analysis Results**: Due to randomization of task order, participants were divided into two groups. The first group (referred to as the “Initial AI Integration” group) experienced AI integration in their workflow before switching to a non-AI workflow. The second group, the “Delayed AI Integration” group, started without AI and later integrated AI into their workflow.
  - **Initial AI Integration Group**: No significant change in job autonomy was observed (**t = 1.11, p = 0.27, d = 0.06**), with a CI of **-0.13 to 0.25**.
  - **Delayed AI Integration Group**: A **significant increase** in job autonomy post-AI integration was found (**t = 2.94, p = 0.004, d = 0.17**), with a CI of **-0.02 to 0.36**.
- Gender Differences.
  - **Male Participants**: A **significant increase** in job autonomy was observed (**t = 2.92, p = 0.004, d = 0.17**), with a CI of **-0.02 to 0.35**.
  - **Female Participants**: No significant change in job autonomy was detected (**t = 1.06, p = 0.29, d = 0.06**), with a CI of **-0.13 to 0.25**.
- **ANCOVA**: After controlling for gender, cultural orientation, and age, AI integration still had a significant effect on job autonomy (**F =  5.95, p =  0.02, η² =  0.012**), indicating that AI integration explained a small portion of the variance in job autonomy beyond the control variables.
- Repeated Measures ANOVA: A significant main effect of AI integration on job autonomy was found (F =  5.06, p =  0.03, ges =  0.002). However, the main effect of cultural orientation was not significant (F =  1.77, p =  0.18, ges =  0.003.
- **Interpretation**: Overall, AI integration had a **positive impact** on job autonomy, contrary to the original hypothesis. The **Delayed AI Integration group** showed a significant increase in job autonomy after AI was introduced, suggesting that delayed exposure to AI might lead to greater perceived autonomy. However, the **Initial AI Integration group** did not experience a significant change, indicating that initial exposure to AI may not have a marked effect on autonomy perception.

**Fig 2:**
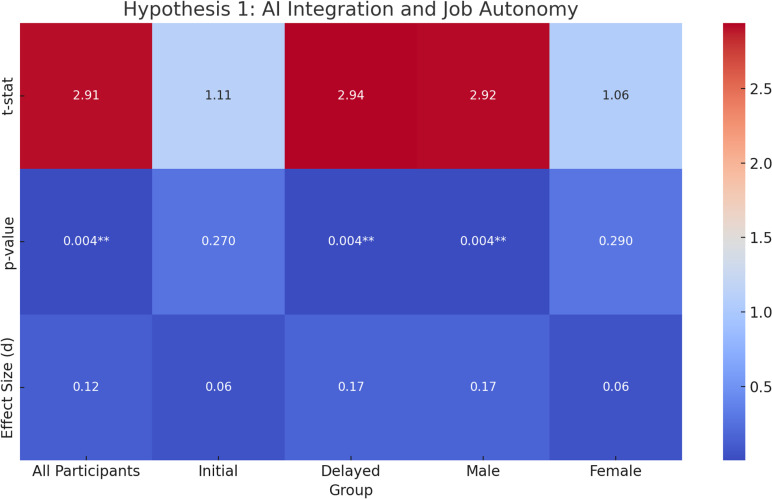
Heat Map of Hypothesis 1 (AI Integration and Job Autonomy) Results. This heat map illustrates the t-statistics (top row), p-values (middle row), and effect sizes (bottom row) for each subgroup tested under Hypothesis 1. The color scale ranges from − 1 (dark blue) to + 3 (bright red), with p < .05 indicated by double asterisks. Warmer colors correspond to larger positive values, cooler colors to smaller or negative values.

Fig 2 presents a heat map summarizing the t-statistics, p-values, and effect sizes for all subgroups under Hypothesis 1.

### Hypothesis 2: AI integration and creative self-efficacy

- **Original Hypothesis**: AI integration positively influences creative self-efficacy.
- **Overall Results**: Across all participants, there was **no significant change** in creative self-efficacy after AI integration (**t =  0.67, p =  0.50, d =  0.02**), with a CI of **-0.11 to 0.16**.
- **Exploratory Analysis Results**: Further analysis revealed gender-specific effects:
  - Male Participants: A marginally significant decrease in creative self-efficacy post-AI integration was found (t = 1.74, p = 0.08, d = 0.08), with a CI of -0.10 to 0.27.
  - **Female Participants**: No significant change in creative self-efficacy post-AI integration (**t = -0.90, p = 0.37, d = -0.04**), with a CI of **-0.23 to 0.16**.
- **ANCOVA**: After controlling for gender, cultural orientation, and age, AI integration had a marginal effect on creative self-efficacy (**F =  3.70, p =  0.06, η² =  0.002**).
- **Repeated Measures ANOVA**: There was **no significant main effect** of time (pre/post-AI) on creative self-efficacy (**F =  1.36, p =  0.24, ges =  0.000**). However, the **main effect of cultural orientation** was significant (**F =  13.78, p =  0.000, ges =  0.028**), suggesting that cultural orientation influences creative self-efficacy post-AI.
- **Interpretation**: The results suggest that **AI integration does not significantly improve creative self-efficacy** across all participants, contrary to the hypothesis. However, the findings highlight a gender-based difference, with male participants showing a marginal decrease in creative self-efficacy, whereas female participants remained unaffected.

**Fig 3:**
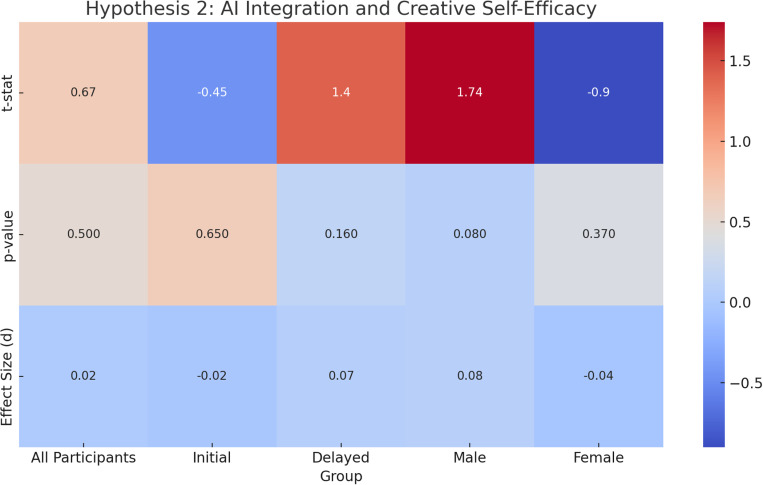
Heat Map of Hypothesis 2 (AI Integration and Creative Self-Efficacy) Results. This heat map illustrates the t-statistics (top row), p-values (middle row), and effect sizes (bottom row) for each subgroup tested under Hypothesis 2. The color scale ranges from − 1 (dark blue) to + 3 (bright red). Warmer colors correspond to larger positive values, cooler colors to smaller or negative values.

Fig 3 presents a heat map summarizing the t-statistics, p-values, and effect sizes for all subgroups under Hypothesis 2.

### Hypothesis 3: cultural orientation and AI integration

- **Original Hypothesis**: Individuals from collectivistic cultures are more receptive to AI integration compared to those from individualistic cultures.
- **Results**:
  - **Job Autonomy**: There was **no significant difference** between individualistic and collectivistic cultures in terms of job autonomy after AI integration (**t = -1.04, p = 0.30, d = -0.10**), with a CI of **-0.30 to 0.09**.
  - **Creative Self-Efficacy**: A **significant difference** was found between individualistic and collectivistic cultures post-AI integration (**t = 3.77, p = 0.000, d = 0.36**), with a CI of **0.17 to 0.55**. Collectivistic cultures showed greater gains in creative self-efficacy.
- **ANCOVA**: After controlling for gender and age, cultural orientation had a **significant effect** on both job autonomy (**F =  4.42, p =  0.04**) and creative self-efficacy (**F =  13.89, p =  0.000**).
- **Repeated Measures ANOVA**: There was a marginal interaction between culture and time for job autonomy (**F =  3.51, p =  0.07, ges =  0.001**), but no significant interaction for creative self-efficacy (**F =  1.18, p =  0.28, ges =  0.000**).
- **Interpretation**: The results indicate that **cultural orientation** influences perceptions of AI integration, especially in the case of **creative self-efficacy**, where participants from collectivistic cultures experienced a more substantial improvement. However, the impact of AI integration on **job autonomy** did not differ significantly between individualistic and collectivistic cultures.

**Fig 4:**
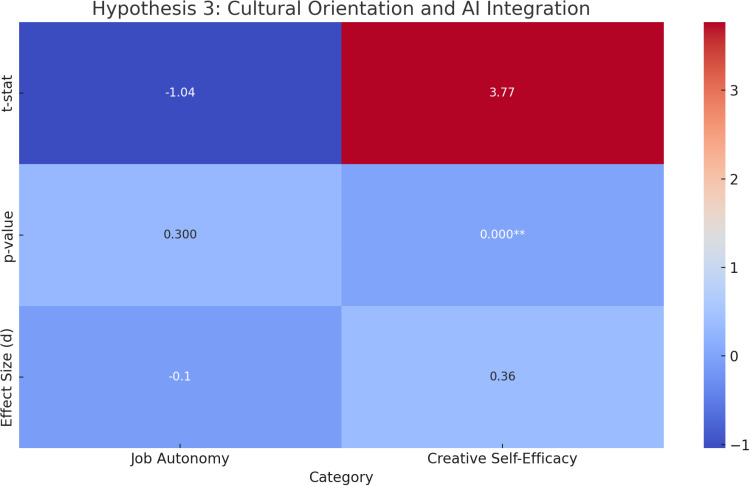
Heat Map of Hypothesis 3 (Cultural Orientation and AI Integration) Results. This heat map illustrates the t-statistics (top row), p-values (middle row), and effect sizes (bottom row) for each subgroup tested under Hypothesis 3. The color scale ranges from − 1 (dark blue) to + 3 (bright red), with p < .05 indicated by double asterisks. Warmer colors correspond to larger positive values, cooler colors to smaller or negative values.

Fig 4 presents a heat map summarizing the t-statistics, p-values, and effect sizes for all subgroups under Hypothesis 3.

Table 1 further summarizes the main statistical findings for all hypotheses, including effect sizes and confidence intervals, offering a consolidated view of how AI integration impacted job autonomy and creative self-efficacy across cultural orientations.

**Table 1:** Summary of statistical analyses for the effects of AI integration on job autonomy and creative self-efficacy including exploratory analyses.

Hypothesis	Analysis	t/F	p	d/η²/ges	95% CI	M(SD), Pre AI	M(SD), Post AI
**Hypothesis 1: AI Integration and Job Autonomy**							
Post vs Pre AI Integration (All Participants)	T-test *(Overall)*	t = 2.91	0.004**	d = 0.12	[0.04, 0.20]	3.73(0.84)	3.83(0.76)
Post vs Pre AI Integration (Initial)	T-test *(Initial)*	t = 1.11	0.27	d = 0.06	[-0.13, 0.25]	3.80(0.81)	3.85(0.74)
Post vs Pre AI Integration (Delayed)	T-test *(Delayed)*	t = 2.94	0.004**	d = 0.17	[-0.02, 0.36]	3.67(0.87)	3.81(0.77)
Post vs Pre AI Integration (Male)	T-test *(Male)*	t = 2.92	0.004**	d = 0.17	[-0.02, 035]	3.71(0.85)	3.85(0.80)
Post vs Pre AI Integration (Female)	T-test *(Female)*	t = 1.06	0.29	d = 0.06	[-0.13, 0.25]	3.76(0.84)	3.80(0.72)
Controlling for cultural orientation, gender and age	ANCOVA *(Overall)*	F = 5.95	0.02*	η² = 0.012	–	–	–
Main Effect of AI Integration	Repeated Measures ANOVA	F = 5.06	0.03*	ges = 0.002	–	–	–
Main Effect of Cultural Orientation	Repeated Measures ANOVA	F = 1.77	0.18	ges = 0.003	–	–	–
**Hypothesis 2: AI Integration and Creative Self-Efficacy**							
Post vs Pre AI Integration (All Participants)	T-test *(Overall)*	t = 0.67	0.50	d = 0.02	[-0.11, 0.16]	4.07(0.72)	4.08(0.78)
Post vs Pre AI Integration (Initial)	T-test *(Initial)*	t = -0.45	0.65	d = -0.02	[-0.21, 0.17]	4.01(0.76)	4.00(0.81)
Post vs Pre AI Integration (Delayed)	T-test *(Delayed)*	t = 1.40	0.16	d = 0.07	[-0.12, 0.26]	4.12(0.68)	4.17(0.74)
Post vs Pre AI Integration (Male)	T-test *(Male)*	t = 1.74	0.08	d = 0.08	[-0.10, 0.27]	4.12(0.70)	4.18(0.72)
Post vs Pre AI Integration (Female)	T-test *(Female)*	t = -0.90	0.37	d = -0.04	[-0.23, 0.16]	4.01(0.75)	3.98(0.83)
Controlling for cultural orientation, gender and age	ANCOVA *(Overall)*	F = 3.70	0.06	η² = 0.002	–	–	–
Main Effect of AI Integration	Repeated Measures ANOVA	F = 1.36	0.24	ges = 0.000	–	–	–
Main Effect of Cultural Orientation	Repeated Measures ANOVA	F = 13.78	0.000***	ges = 0.028	–	–	–
**Hypothesis 3: Cultural Orientation and Perception of AI Integration**							
Individualistic vs Collectivistic	T-test *(Job Autonomy)*	t = -1.04	0.30	d = -0.10	[-0.30, 0.09]	†3.81(0.80), ‡3.62(0.89)	†3.86(0.72), ‡3.78(0.81)
Individualistic vs Collectivistic	T-test *(Creative Self-Efficacy)*	t = 3.77	0.000***	d = 0.36	[0.17, 0.55]	†4.01(0.71), ‡4.15(0.73)	†3.97(0.82), ‡4.24(0.70)
Controlling for gender and age	ANCOVA *(Job Autonomy)*	F = 4.42	0.04*	η² = 0.001	–	–	–
Controlling for gender and age	ANCOVA *(Creative Self-Efficacy)*	F = 13.89	0.000***	η² = 0.021	–	–	–
Cultural Orientation x AI Integration (Interaction)	Repeated Measures ANOVA *(Job Autonomy)*	F = 3.51	0.07	ges = 0.001	–	–	–
Cultural Orientation x AI Integration (Interaction)	Repeated Measures ANOVA *(Creative Self-Efficacy)*	F = 1.18	0.28	ges = 0.000	–	–	–

## Discussion

In this section, we will discuss the findings of our study in five key points: the relationship between AI integration and job autonomy, the influence of AI integration on creative self-efficacy, the impact of cultural orientation on perceptions of AI integration, gender-specific effects of AI integration, and the effects of the order of AI integration.

Contrary to Hypothesis 1, which posited that AI integration would negatively influence perceived job autonomy, our findings demonstrated a significant increase in job autonomy following AI integration. This suggests that AI, rather than undermining autonomy, may provide employees with greater discretion over high-level tasks by automating routine or mundane activities [34,35]. The exploratory analysis further revealed that the Delayed AI Integration group experienced a significant increase in job autonomy post-AI. However, the Initial AI Integration group did not show a significant change, indicating that exposure to AI early in the workflow might not have as profound an effect on perceived autonomy. This suggests that the sequence in which AI is introduced plays a critical role in shaping its perceived impact [11,36]. The contrast effect could explain why the Delayed AI group felt a more pronounced increase in autonomy, as the shift from a non-AI environment to an AI-enhanced one may highlight the advantages of AI [21]. Additionally, after controlling for gender, cultural orientation, and age, AI integration continued to have a significant effect on job autonomy, suggesting that even when these variables are accounted for, AI’s influence on autonomy remains. These findings point to the complexity of AI’s impact, emphasizing the importance of context, timing, and user familiarity.

Hypothesis 2 suggested that AI integration would positively influence creative self-efficacy. However, across all participants, no significant change in creative self-efficacy was observed post-AI integration. This aligns with literature suggesting that simply introducing AI may not be enough to enhance creative self-efficacy; organizational practices that foster innovation are essential for maximizing the benefits of AI [19]. Our exploratory analysis uncovered gender-specific effects. Male participants experienced a marginal decrease in creative self-efficacy following AI integration. In contrast, female participants did not exhibit any significant change. These findings indicate that AI may have a differential impact on creative self-efficacy based on gender. While the effects observed among males were small, the marginal significance suggests that further investigation is warranted. One possible explanation is that societal norms around control and independence may lead men to feel that AI undermines their creative confidence by automating decision-making tasks traditionally seen as markers of competence [24].

Hypothesis 3 posited that individuals from collectivistic cultures would be more receptive to AI integration than those from individualistic cultures. While our results showed no significant difference in job autonomy between individualistic and collectivistic cultures, there was a significant difference in creative self-efficacy, with collectivistic cultures showing a more substantial improvement post-AI. These findings suggest that cultural orientation plays a more prominent role in shaping perceptions of creative self-efficacy than job autonomy. In collectivistic cultures, where group collaboration and interdependence are highly valued, AI may be viewed as a tool that enhances collective creativity [30]. This contrasts with individualistic cultures, where AI’s role in supporting autonomy may be less pronounced [15]. However, the lack of a significant cultural difference in job autonomy suggests that AI’s perceived impact on autonomy may transcend cultural boundaries, potentially due to its role in augmenting decision-making regardless of individual or collective orientations [26,24].

Our exploratory analysis revealed a significant impact of AI integration on creative self-efficacy among male participants but not among female participants. This observation could be attributed to prevailing gender norms that emphasize control and independence more strongly in men than in women [37]. Such norms may predispose men to perceive the introduction of AI integration—a technology that automates tasks and can make decisions—as a more direct threat to their professional autonomy [22,38]. On the other hand, female participants did not exhibit significant changes in their perception of autonomy. This difference might be linked to gender-specific expectations and socialization processes, where women might either view technological aids in a less threatening manner or have different expectations about employee autonomy and control in the workplace [37]. Moreover, the relative stability in women’s perceptions from the outset suggests that their response to AI integration could be less about a perceived loss of autonomy and more about how these tools can be utilized to enhance job performance without undermining their role [22].

The order of AI integration in the workflow significantly affected job autonomy. Participants initially experiencing a work environment without AI showed a more pronounced decrease in perceived autonomy when AI was later introduced. This can be explained by the contrast effect—a psychological phenomenon where introducing a new factor is more starkly felt against the backdrop of a previous condition without that factor [38]. In contrast, participants starting with AI in their workflows and later experiencing its removal did not exhibit significant changes in their perceptions. This group’s initial adaptation to AI might have moderated their perceptions from the outset, setting a different baseline where AI’s presence was normative [8]. The removal of AI might not have been perceived as an enhancement of autonomy but rather as a return to a less preferred state without the assistance of AI. This suggests that initial exposure to AI may help to establish familiarity and comfort with the technology, making subsequent changes less disruptive. Familiarity with AI plays a crucial role in shaping perceptions and attitudes toward its integration. Research indicates that familiarity can reduce perceived threats and increase acceptance of new technologies [34]. When employees are introduced to AI from the start and gradually build their understanding and skills around its usage, they are more likely to see it as a supportive tool rather than an intrusive one [4]. This progressive exposure leads to smoother transitions and higher comfort levels with AI, mitigating negative perceptions related to autonomy and control [14].

## Limitations and future research

Our study has provided valuable insights into the complex relationship between cultural orientation, AI integration, job autonomy, and creative self-efficacy. However, it is essential to acknowledge the limitations of our research and propose areas for future research that can build upon these findings. In this section, we will discuss the limitations and recommendations in six key points.

First, a primary limitation in our study is the small effect sizes observed, which might stem from the controlled experimental setting. Such environments often fail to fully capture the complexities of real-world workplaces, potentially limiting our ability to observe the extensive impacts of AI integration on employee perceptions [19]. To address this and enhance the generalizability of our findings, future research should consider conducting real-life field studies that observe participants in their everyday work environments over extended periods. These studies could provide ecologically valid insights into the long-term effects of AI on job autonomy and creative self-efficacy, allowing for a better understanding of how AI integration interacts with the dynamic and multifaceted nature of real-world work settings. Additionally, expanding the scope to include a broader range of fields—such as manufacturing, healthcare, and education—would allow researchers to explore how attitudes towards AI and autonomy might vary across different occupational contexts. This broader approach could help identify specific factors that influence the effectiveness of AI integration and provide more actionable recommendations for implementing AI across diverse workplace environments.

Second, although our research focused on the UK and Mexico, providing insights into specific cultural contexts, it also limits the breadth of cultural perspectives explored. Expanding future research to include comparative studies across a broader range of countries and cultures could help identify cross-cultural patterns and variations in AI perceptions. It is also crucial to include diverse demographic factors within these studies, such as age and gender, to assess how these variables interact with cultural orientations in shaping AI adoption and its impacts. For instance, different age groups and genders may have varying degrees of receptivity towards AI, influenced by cultural norms and personal experiences [19]. A global comparative analysis that accounts for these factors might assess regional or national differences in AI adoption and how cultural orientations, alongside economic, regulatory, and technological factors, shape these trends [17]. This would enhance our understanding of how different cultural contexts affect AI integration and how demographic factors further modulate these effects.

Third, our study’s participant age ranges were carefully chosen to represent the workforce demographics of Mexico and the UK, reflecting each country’s typical labour force. In Mexico, with its younger workforce and early job market entry, the 18-54 year age range aligns with national statistics [17]. In contrast, the UK workforce, characterized by a significant proportion of older employees and longer career spans, is represented by the broader 18-65 year age range. Exploring additional age brackets or career stages is crucial for understanding how AI impacts might impact different stages of a career. A more diverse age sample could reveal differences in how various age groups experience and adapt to AI, thereby offering a more comprehensive view of its effects on employee perceptions.

Fourth, while our quantitative approach provided structured insights, integrating qualitative research methods such as in-depth interviews or focus group discussions could offer a deeper understanding of the cultural factors influencing AI integration. These qualitative methods would allow participants to express their perspectives and experiences more freely, revealing personal narratives and group dynamics that might remain hidden in survey data. This approach would enrich our understanding of cultural facilitators or barriers to AI adoption and inform more culturally sensitive and inclusive AI integration strategies.

Fifth, our study did not extensively explore alternative methods of measuring receptivity to AI, such as trust in AI systems. Future research should investigate how biases embedded within AI systems could perpetuate or exacerbate workplace inequalities, influencing employee trust and acceptance of these technologies [39]. Additionally, building trust in AI technologies through transparent communication about AI functionalities, decision-making processes, and protective measures for employee data is crucial [40]. Exploring these aspects can provide a more comprehensive understanding of employees’ receptivity to AI, beyond job autonomy and creative self-efficacy, and help organizations implement AI in ways that foster trust and minimize resistance.

Finally, considering the dynamic nature of technology and work environments, longitudinal studies tracking the evolution of AI integration over time would be beneficial. These studies could observe how perceptions and impacts change as employees become more accustomed to AI in their workflows, paying attention to how these changes differ over time. Additionally, researching and testing interventions or best practices for culturally sensitive AI implementation could guide organizations in optimizing AI strategies within diverse workforces. Such research could also explore how interventions can address specific concerns related to gender differences or masculinity-femininity traits and their impact on the acceptance and effectiveness of AI technologies [37].

By addressing these limitations and exploring the proposed areas for future research, subsequent studies can advance our understanding of how AI technologies interact with cultural and demographic dynamics in the workplace. This ongoing research is crucial for developing effective strategies that leverage AI’s potential while accommodating the diverse needs and perspectives of the global workforce.

## Conclusion

This study provides crucial insights into the multifaceted impacts of AI integration on job autonomy and creative self-efficacy, influenced by cultural orientation. First, we observed a significant overall decrease in job autonomy following AI integration, supporting our initial hypothesis that AI could negatively impact perceived autonomy. These results suggest that AI’s role in standardizing and automating tasks may lead to a perceived loss of control and discretion in work-related activities. Second, contrary to our hypothesis that AI would enhance creative self-efficacy, our findings revealed no overall improvement in creative self-efficacy post-AI integration. Notably, gender-specific analyses revealed that male participants experienced a significant decrease in creative self-efficacy following AI integration, whereas female participants’ perceptions remained largely unchanged. This suggests that while AI may not uniformly boost creative abilities, it could have a detrimental impact on male employees’ perceived creative self-efficacy, highlighting the importance of considering gender differences in evaluating technological impacts. Third, our study confirmed that cultural orientation significantly affects perceptions of AI integration, particularly regarding job autonomy. Participants from individualistic cultures, such as those in the UK, reported a more pronounced decrease in job autonomy compared to those from collectivistic cultures like Mexico. This supports our hypothesis that cultural attitudes toward technology and collaboration shape how AI is perceived and experienced in terms of autonomy. Although the effect on creative self-efficacy was not statistically significant, there was a trend indicating that collectivistic cultures might perceive some enhancement in creative self-efficacy, suggesting a potentially positive view of AI’s role in supporting collective success. Overall, these findings illustrate the intricate and variable impacts of AI integration on different cultural and demographic groups. The study’s limitations, including small effect sizes and the controlled experimental environment, highlight the need for further research in real-world settings.

## Supporting Information

S1 File Study Data. (XLSX)
